# Catatonia in *ICD-11*

**DOI:** 10.1186/s12888-025-06857-6

**Published:** 2025-04-18

**Authors:** Jonathan P. Rogers, Jo Ellen Wilson, Mark A. Oldham

**Affiliations:** 1 Division of Psychiatry, University College London, London, UK; 2 Veterans Affairs, Geriatric Research, Education and Clinical Center, Tennessee Valley Healthcare System, Nashville, TN USA; 3 Department of Psychiatry and Behavioral Sciences, Vanderbilt University Medical Center, Nashville, TN USA; 4 Department of Psychiatry, University of Rochester Medical Center, Rochester, NY USA

**Keywords:** Catatonia, Catatonic schizophrenia, ICD-11, Classification

## Abstract

In the *International Statistical Classification of Diseases and Related Health Problems Version 11* (*ICD-11*), the diagnostic criteria for catatonia have been extensively revised. We provide context for these changes beginning with a brief history of how catatonia has been conceptualized and a description of how the criteria for catatonia have changed across *ICD* versions. We also compare *ICD-11* with the criteria in the latest *Diagnostic and Statistical Manual of Mental Disorders* (i.e., *DSM-5-TR*), consider clinical implications of the changes in *ICD-11*, and highlight conceptual areas in need of further development.

Described in 1874 by Karl Kahlbaum, catatonia was subsequently subsumed into the Kraepelinian concept of dementia praecox. This was reflected in versions of *ICD* up to *ICD-9*, which considered catatonia exclusively as a form of schizophrenia. *ICD-10* introduced the diagnosis of organic catatonic disorder but did not incorporate the growing evidence that catatonia can occur in psychiatric conditions such as mood and autism-spectrum disorders.

*ICD-11* conceptualizes catatonia as an independent disorder with a common clinical phenotype regardless of associated condition, if present. In *ICD-11*, catatonia diagnosis requires at least three clinical features from the following categories: decreased, increased or abnormal psychomotor activity. These features may come from any combination of the categories, but only one from the increased psychomotor activity category should be counted. The four catatonia diagnoses in *ICD-11* are catatonia associated with another mental disorder, catatonia induced by substances or medications, secondary catatonia syndrome and catatonia, unspecified. This expanded view of catatonia more closely resembles *DSM-5-TR*, which also recognizes catatonia associated with several psychiatric and general medical conditions. *ICD-11* also offers guidance on distinguishing catatonia from similar behavioural features of other conditions, such as psychomotor retardation in depression, delirium, and factitious disorder.

This new classification stands to improve recognition of catatonia and our hope is that it may lead to a growing awareness of the wide range of conditions associated with it. Ultimately, a better understanding of catatonia should contribute to improved outcomes as clinicians offer treatments both for catatonia itself as well as tailored treatments for its broad range of associated conditions.

## Background

Catatonia is one of the psychiatric disorders that has undergone the greatest change in the transition to the *International Statistical Classification of Diseases and Related Health Problems*,* Version 11*, commonly known as *ICD-11* [1]. *ICD-11* recognises catatonia as an independent diagnostic category with four possible diagnoses: catatonia associated with another mental disorder, catatonia induced by substances or medications, secondary catatonia syndrome, and catatonia, unspecified [2]. 

In this article, we briefly describe the history of catatonia, how this disorder has been represented in previous versions of *ICD*, and its conceptual development in *ICD-11*. We then consider how this compares to *DSM-5-TR*, the clinical implications for the new *ICD-11* catatonia criteria and outstanding diagnostic issues that should be addressed.

## History

Neuropsychiatric conditions that are consistent with contemporary descriptions of catatonia were characterised as early as the first century AD in India, as well as in the Greco-Roman medical literature [3, 4]. Soranus of Ephesus was likely describing comorbid catatonia and delirium when he wrote, ‘Quiet or loud laughter, singing or a state of sadness, silence, murmuring, crying, or a barely audible muttering to one’s self; or such a state of anger that the patient jumps up in a rage and can scarcely be held back, is wrathful at everyone, shouts, beats himself... or seeks to hide in fear, or weeps, or fails to answer those who speak with him, while he speaks not only with those who are not present but with the dead.’ [5] Van Druppen and Sienaert [6] quote the English physician Philip Barraugh vividly describing “a sodaine detention and taking both of mind and body, both sense and moving being lost, the sicke remaining in the same figure of bodie wherin he was taken, whither he sit or lye, or stand, or whiter his eyes be open or shut”. However, interest in movement disorders from a neurological and psychiatric perspective intensified in the 19th century and terms such as ‘*melancholia attonita’*, ‘catalepsy’, ‘stereotypy’, ‘mannerism’, ‘negativism’ and ‘*stupidité*’ or ‘stupor’ became used to encapsulate at least some of the modern concept of catatonia [7]. Conflicting schools of thought held movement disorders to be either secondary to an abnormal mental state or involuntary products of abnormal brain activity, but it was the German neurologist and psychiatrist Wilhelm Griesinger who started to place individual movement disorders in either one or other of these categories [8]. 

In 1874, Karl Ludwig Kahlbaum, a German psychiatrist, was the first to systemize catatonia, giving it its current name [9]. In his monograph entitled ’*Die Katatonie oder das Spannungsirresein*’, Kahlbaum described an assortment of case histories of 26 patients with catatonia stemming from a variety of psychiatric, neurologic, medical and infectious causes. Kahlbaum recognised that catatonia frequently included alternation between stupor and excitement [10], but his particular interest was the longitudinal course of mental disorders, which led him to postulate a four-stage model of progression in catatonia, passing through melancholia, manic rage, stuporous melancholia and (on occasion) terminal dementia [11]. His goal was to establish the same neuropathological correlation for catatonia as had been done for the general paresis of the insane [7]. 

Emil Kraepelin was also interested in disease outcome and, conceiving catatonia to end in widespread cognitive dysfunction, he included it in his concept of dementia praecox [11]. However, unlike Kahlbaum, who considered that motor signs could be primary manifestations of mental disorders, Kraepelin had an exclusively psychological interpretation of such signs [11]. Carl Wernicke, better known for Wernicke’s encephalopathy and Wernicke’s aphasia, meanwhile broadened Kahlbaum’s work out to a wider integration of motor signs in the understanding of mental disorders, which was termed ‘*Motilitätpsychosen’* [8]. 

## Catatonia up to *ICD-10*

Catatonia was not used in any formal diagnostic systems prior to *ICD-6* (1948). The status of catatonia in *ICD-6* to *ICD-11* is shown in Table 1. Up through *ICD-9* (1975), catatonia is essentially the same across versions of the *ICD* with a single diagnostic code representing a catatonic form of schizophrenia. The substantive change in *ICD-10* (1990) was to add a diagnostic entity termed ‘Organic catatonic disorder’ [12]. A year after *ICD-9* was published, two significant papers from the USA challenged the notion that catatonia occurred exclusively in schizophrenia. The first, by Abrams and Taylor, was a prospective study of consecutive hospital admissions in New York City, which identified 55 patients with at least one catatonic feature, of whom 39 (71%) ultimately received a research diagnosis of an affective disorder, 9 (16%) an organic brain syndrome, 3 (5%) a reactive psychosis and only 4 (7%) schizophrenia [13]. The second was a narrative review by Gelenberg that examined the various psychiatric (including affective and neurotic), neurological and general medical conditions that may manifest catatonic signs, concluding that clinicians need to be aware of serious ‘organic’ illnesses that might underlie catatonia [14]. 

Accordingly, *ICD-10* inserted ‘Organic catatonic disorder’ into its section on ‘Organic, including symptomatic, mental disorders’ to account for catatonia appearing in neurological and general medical conditions. It is unclear why the *ICD-10* authors incorporated the evidence on catatonia in neuro-medical conditions but not in affective disorders.

A second idiosyncrasy of catatonia in *ICD-10* is that the criteria for catatonic schizophrenia are different from and more restrictive than those for organic catatonic disorder. For organic catatonic disorder, according to *ICD-10*, the patient may present with one of the following: stupor, excitement or rapidly and unpredictably shifting between hypo- and hyperactivity. Stereotypies, waxy flexibility and impulsive acts are listed among other catatonic phenomena that may increase the confidence in the diagnosis. By way of contrast, diagnosis of catatonic schizophrenia required at least one of the following features: stupor, excitement, posturing, negativism, rigidity, waxy flexibility, command automatisms or perseveration.

A third consideration with catatonia in *ICD-10* is the requirement for only a single catatonic feature for diagnosis in association with schizophrenia. Subsequent analyses have found this to be over-inclusive, given that no one clinical feature is pathognomonic for catatonia [15, 16]. For instance, excitement may occur in mania, stimulant intoxication, sedative-hypnotic withdrawal, and acute psychosis. Despite this criticism, it remains unclear to these authors what minimal set of clinical features is required to suggest catatonia.

Fourth, catatonia duration in *ICD-10* is also problematic. Whereas the minimum duration of organic catatonic disorder is unspecified, diagnosing catatonic schizophrenia requires that a person first meet the criteria for schizophrenia including being symptomatic for at least one month. Given that catatonia has been associated with various medical complications (including venous thromboembolism, sepsis and cardiac dysrhythmia) [17] and several studies have suggested that earlier treatment is associated with better outcomes [18–21], it would be inexcusable to delay clinical diagnosis for up to a month.

Finally, *ICD-10* did not allow for a diagnosis of idiopathic catatonia. Patients with a primary recurring catatonia were studied in the middle of the 20th century, notably by Rolf and Leiv Gjessing [22]. Recent studies have also found evidence of cases of idiopathic catatonia that respond to benzodiazepines and electroconvulsive therapy (ECT) similar to other forms of catatonia [23–25]. 

**Table 1:** Catatonia diagnoses in *ICD-6* to *ICD-11*

	ICD-6	ICD-7	ICD-8	ICD-9	ICD-10	ICD-11
Date	1948	1955	1965	1975	1990	2019
Schizophrenia, catatonic type	300.2 - Schizophrenia disorders (dementia praecox), catatonic type	300.2 - Schizophrenia disorders (dementia praecox), catatonic type	295.2 - Schizophrenia, catatonic type	295.2 - Schizophrenic psychoses, catatonic type*	F20.2 - Catatonic schizophrenia	
Medical catatonia					F06.1 - Organic catatonic disorder	6E69 - Secondary catatonia syndrome
Catatonia induced by substances or medications						6A41 - Catatonia induced by substances or medications
Catatonia associated with another mental disorder						6A40 - Catatonia associated with another mental disorder
Catatonia unspecified						A4Z - Catatonia, unspecified

*ICD - International statistical classification of diseases and related health problems*

*Consistent with other types of schizophrenia, *ICD-9* recognised six subclassifications for the catatonic type: 295.20, Unspecified; 295.21, Subchronic; 295.22, Chronic; 295.23, Subchronic with acute exacerbation; 295.24, Chronic with acute exacerbation; 295.25, In remission

## Catatonia phenotype in *ICD-11*

The changes to catatonia in *ICD-11* (2019) were driven by the goal to revise *ICD-10* in line with the latest evidence, not only on catatonia but also as a response to the anticipated removal of schizophrenia subtypes. Leading up to *ICD-11*, it had been increasingly appreciated that schizophrenia’s subtypes are longitudinally unstable and artificially dichotomise overlapping clinical features [26]. Simply removing catatonic schizophrenia from *ICD* would have left catatonia orphaned only as organic catatonic disorder in *ICD-11*.

Instead, *ICD-11* consolidated catatonia under a single diagnostic category [2]. Included are fifteen clinical features of catatonia independent of associated condition, which closely parallels the 12 features included in *DSM-5-TR* (Table 2). The clinical features must be present for at least several hours and either result in significant functional impairment or be severe enough to cause medical complications.

**Table 2:** Features of catatonia in *ICD-11* and *DSM-5-TR*

Clinical feature	Description*	ICD-11	DSM-5-TR
Decreased psychomotor activity			
Staring	Fixed gaze, decreased blinking	X	
Ambitendency	Appears ‘motorically stuck’ in indecision	X	
Negativism	Opposing or behaving contrary to requests	X	X
Stupor	No or markedly reduced psychomotor activity	X	X
Mutism	No or very little verbal response (excludes other conditions affecting speech)	X	X
Increased psychomotor activity			
Extreme hyperactivity	May include agitation or extreme emotions	X	X
Impulsivity	Sudden, unprovoked, inappropriate behavior		
Combativeness	Undirected striking out against others		
Abnormal psychomotor activity			
Grimacing	Odd or distorted facial expressions	X	X
Mannerisms	Odd but purposeful movements	X	X
Posturing	Spontaneous maintenance of a posture held against gravity	X	X
Stereotypy	Repetitive, non-goal-directed motor activity	X	X
Rigidity	Resistance with increased muscle tone	X	
Echolalia	Mimicking examiner’s speech	X	X
Echopraxia	Mimicking examiner’s movements		X
Verbigeration	Repetition of words, phrases, or sentences	X	
Waxy flexibility^†^	Slight, even resistance to positioning	X	X
Catalepsy	Passive induction of a posture held against gravity	X	X

*ICD - International statistical classification of diseases and related health problems*

*DSM - Diagnostic and statistical manual of mental disorders*

*Abridged

^†^The Bush-Francis Catatonia Rating Scale defines waxy flexibility categorically as initial resistance before allowing oneself to be repositioned

The 15 phenotypic criteria of catatonia are divided into three motoric categories—decreased, increased, or abnormal psychomotor activity—broadly reflecting results of research that has identified hypokinetic, hyperkinetic, and parakinetic types, respectively [27, 28]. Of uncertain significance is the unequal distribution of criteria across these three motoric categories, with nine criteria under abnormal, 5 under decreased, and 1 aggregated criterion under increased psychomotor activity. One implication of the distribution of criteria in *ICD-11*, as with *DSM-5-TR*, is that purely hypokinetic and parakinetic types of catatonia may be diagnosed whereas hyperkinetic catatonia will require additional features from these other two psychomotor types.

Four catatonia diagnoses are recognised in *ICD-11*. Each of the three specified catatonia diagnoses has additional diagnostic criteria as shown in Table 3: catatonia associated with another mental disorder, catatonia induced by substances or medications and secondary catatonia syndrome. A diagnosis of catatonia, unspecified is included as a residual category if criteria for none of the three specified diagnoses are satisfied.

**Table 3:** Criteria for specific catatonia diagnoses in *ICD-11*

Code	Parent category	Diagnosis	Additional diagnostic criteria*
6A40	Catatonia	Catatonia associated with another mental disorder	• Catatonia develops in the context of another mental disorder. • Clinical features are not fully accounted for by delirium, effects of a medication or substance, or a primary movement disorder. • Clinical features are sufficiently severe to be a focus of clinical attention.
6A41	Catatonia	Catatonia induced by substances or medications	• Catatonia develops during or soon after intoxication with or withdrawal from a psychoactive substance or medication. • Intensity or duration of catatonic features is substantially greater than other symptoms that are characteristic of the specified substance. • The substance is known to cause catatonia. • Clinical features are not fully accounted for by delirium, another mental disorder, or a medical condition. • Clinical features are sufficiently severe to be a focus of clinical attention.
6E69	Secondary mental or behavioural syndromes associated with disorders or diseases classified elsewhere	Secondary catatonia syndrome	• Clinical features are judged to be the direct pathophysiological consequence of a medical condition. • The medical condition is known to cause catatonia. • The course of catatonia is consistent with causation by the medical condition • The clinical features are not fully accounted for by delirium, another mental disorder, the effects of a medication or substance or a primary movement disorder. • Clinical features are sufficiently severe to be a focus of clinical attention.
6A4Z	Catatonia	Catatonia, unspecified	Residual category

*ICD - International statistical classification of diseases and related health problems*

*Paraphrased criteria

*ICD-11* offers the first set of catatonia diagnostic criteria in which delirium does not exclude catatonia by default. Instead, *ICD-11* requires only that the catatonic features not be “fully accounted for” by delirium or the effects of a medical condition, medication, or substance. Delirium with catatonic features is now increasingly recognised in the literature [29, 30]. There are also important clinical implications. For example, benzodiazepines– the standard of care for catatonia– can worsen delirium, whereas antipsychotics– commonly used to manage neuropsychiatric disturbances in delirium– can precipitate catatonia [31, 32]. In individuals with delirium, the presence of catatonia is associated with viral and autoimmune encephalitis, which should prompt specific antiviral or immunomodulatory therapies [33]. Each of the four catatonia diagnoses may be specified regarding autonomic abnormalities, which include elevated or reduced body temperature, blood pressure, and heart rate. As with other exclusionary comments, vital sign abnormalities cannot be “fully accounted for” by a comorbid medical condition. Recognizing autonomic abnormalities in association with catatonia is imperative because the co-occurrence of catatonia and autonomic abnormalities—a condition called malignant catatonia—is potentially fatal, especially without urgent intervention [34]. It is also important to recognize that both catatonia associated with primary mental conditions and secondary catatonia can present as malignant catatonia.

Careful consideration was given in *ICD-11* to the temporal aspects of the condition. Catatonia can be acute or chronic. Its course can fluctuate, and there can be alternation between stupor and excitement [10, 35–37]. There is some evidence that catatonia with a shorter duration responds better to benzodiazepines [19–21] and one randomized controlled trial of lorazepam for chronic catatonia found it to be ineffective [38], suggesting that duration of catatonia may be important mechanistically. Ultimately, given this heterogeneity, *ICD-11* does not specify a minimum duration, stating that ‘symptoms typically last for at least several hours but can persist much longer’. It acknowledges that for some more severe clinical features, a duration as short as 15 minutes may be sufficient to qualify.

Importantly, *ICD-11* notes that catatonia ‘may occur throughout the entire life span’. This reflects evidence that catatonia can occur in children and adolescents, with cases reported in individuals as young as five years [39]. Likewise, catatonia is an important differential diagnosis in older adults and a lack of recognition has in the past resulted in misdiagnosis as conditions such as stroke, dementia or coma [31, 40, 41]. 

## Boundaries with other disorders and conditions in *ICD-11*

Compared with preceding versions of *ICD*, *ICD-11* contains more advice for clinicians on diagnosing these conditions. In particular, accompanying the diagnostic criteria for catatonia are considerations regarding the boundary of catatonia with several other disorders, which it considers to be differential diagnoses that may present similarly to catatonia, as shown in Table 4. It is worth noting that the depressive, manic and mixed episodes may appear in primary mood disorders and in schizoaffective disorder. Moreover, a phenomenon like psychomotor retardation is also sometimes observed in mania and may be termed ‘inhibited mania’ [42]. This section may have additional utility for establishing a differential diagnosis. However, largely owing to limitations in the published literature, *ICD-11* provides limited advice on distinguishing catatonia from neurological conditions such as movement disorders, speech disorders and functional neurological disorders [28, 43]. Particular difficulties can arise with conditions such as stiff person syndrome, progressive encephalomyelitis with rigidity and myoclonus and Parkinson’s disease, which feature motor rigidity as well as marked alterations in mental state [28]. Malingering and factitious disorder, grouped together in *ICD-11*, do have a distinct history and theoretical framework, even if distinguishing them in clinical practice is difficult [44]. Catatonia also has a complex and poorly defined relationship with functional neurological disorder, formerly known as ‘conversion disorder’ [45–48]. 

The boundary with neuroleptic malignant syndrome (NMS) deserves particular attention because of the controversy it has provoked in the literature. At a minimum, catatonia is a risk factor for NMS. In one case-control study of 12 patients with NMS and 24 antipsychotic-treated controls, matched for age, sex, diagnosis and admission date, 4 of the cases had catatonia compared to none of the controls [49]. In a more recent cohort study of 1719 patients with schizophrenia, the odds ratio for NMS in those with catatonic stupor was 87.0 (95% CI 25.3–462.8) compared to those without catatonic stupor [17]. Autonomic instability differentiates NMS from non-malignant catatonia, but distinguishing malignant catatonia from NMS is difficult [50]. In one study of 16 patients who met DSM-IV criteria for NMS, 15 of them also met DSM-IV criteria for organic catatonia with the severity of NMS correlating with the number of catatonic signs [51]. However, a review of 490 case reports in the literature found that– while clinical features were not entirely specific for diagnoses of NMS or catatonia– there were 11 variables that had some discriminatory power: for example diaphoresis and fever were more common in NMS, whereas posturing and stereotypy were more characteristic of catatonia [52]. At a theoretical level, it has been argued that a similar interaction of GABA-ergic, dopaminergic, serotonergic and glutamatergic systems is implicated in both conditions [53], although the level of evidence for the involvement of certain neurotransmitter systems in catatonia has been questioned [54]. A possible middle position is to argue that antipsychotic-induced catatonia and neuroleptic malignant syndrome exist on a spectrum, as illustrated by one study of 18 patients with antipsychotic-induced catatonia, which found a continuum from simple antipsychotic-induced catatonia without delirium or autonomic involvement at one end, ranging through to full neuroleptic malignant syndrome at the other [55]. The detailed study of boundary conditions and medical mimics of catatonia must be undertaken but is outside the scope of *ICD-11*.

**Table 4:** Other disorders and conditions for which *ICD-11* specifies boundaries with catatonia

Diagnosis	Specified other disorders and conditions
Catatonia associated with another mental disorder	• Psychomotor retardation in depressive or mixed episodes • Psychomotor agitation in depressive, manic or mixed episodes • Neuroleptic malignant syndrome • Serotonin syndrome • Malingering or factitious disorder
Catatonia induced by substances or medications	• Other types of catatonia • Delirium due to disease classified elsewhere • Neuroleptic malignant syndrome • Serotonin syndrome • Malingering or factitious disorder
Secondary catatonia syndrome	• Delirium due to diseases classified elsewhere • Neuroleptic malignant syndrome • Serotonin syndrome • Malingering or factitious disorder

*ICD - International statistical classification of diseases and related health problems*

## Comparison with *DSM-5-TR*

The changes in *ICD-11* broadly parallel recent changes in *DSM* (Table 2). In addition to the long-described catatonic subtype of schizophrenia, *DSM-IV* introduced catatonia due to a general medical condition and a catatonia specifier for major mood disorders. Subsequently, *DSM-5* added a diagnosis of catatonic disorder not otherwise specified and allowed for diagnosis of catatonia in association with neurodevelopmental disorders. Perhaps the most momentous advance introduced by *DSM-5* was the consolidation of a common catatonic phenotype that applied both to primary and secondary (medical) presentations.

*ICD-11* incorporates each of these prior conceptual developments but goes even further. In addition to introducing a diagnosis for catatonia induced by substances or medications, *ICD-11* gives catatonia its own diagnostic category on the same hierarchical level as mood disorders or neurodevelopmental disorders. This elevation in status had been considered for *DSM-5*, with the goal to improve awareness of the syndrome and to affirm that it is not restricted to schizophrenia [56]. However, *DSM-5* did not give catatonia independent status for a few reasons: the conditions underlying catatonia are more stable over time than catatonia itself; the promotion of catatonia would increase apparent comorbidity; and the treatment of catatonia can vary according to the underlying condition [56]. *DSM-5-TR* has not made substantive changes to the definition of catatonia [57]. 

## Clinical implications of *ICD-11* for catatonia

Catatonia is broadly under-diagnosed in clinical practice [58]. It is hoped that promoting it as an independent condition will emphasise its importance. If catatonia is understood merely as a specifier, clinicians are likely to view diagnosing catatonia as something optional within psychiatric formulation. Its new diagnostic classification as a stand-alone condition in *ICD-11* makes a positive case for independently recognising and treating catatonia. This distinction also highlights the need for a unified treatment approach for catatonia, which often requires the use of a benzodiazepine, electroconvulsive therapy (ECT), or both.

Next, the expanded range of diagnoses that can be coded in association with catatonia should also improve recognition of the spectrum of associated conditions. *ICD-10* permitted only organic catatonic disorder and catatonic schizophrenia, likely leading clinicians to overlook other psychiatric conditions. There is strong evidence that catatonia can also present in mood disorders [13, 59], and postpartum psychosis [60–62]. Research in recent years has highlighted the occurrence of catatonia in neurodevelopmental and neurogenetic conditions. A systematic review found that catatonia has a prevalence of 10.4% (95% CI 5.8–18.0%) in autism spectrum disorder (ASD), predominantly in males [63]. The diagnostic issue here is that certain catatonic features (such as stereotypies or echolalia) are present as part of ASD, so it has been recommended to subtract ‘baseline’ scores on catatonia rating scales from the current score in order to establish a true catatonic deterioration [64]. Several genetic abnormalities have been identified in association with catatonia, largely in case reports and small case series [65–68]. The size of these studies makes it difficult to know whether these represent true causal relationships, but a link to Phelan-McDermid syndrome [65, 69, 70] and Down’s syndrome [71, 72] has been reported and may have implications for genetic testing in selected individuals. *ICD-11* encourages clinicians to evaluate for these and other conditions once catatonia has been identified.

Additionally, separating catatonia from schizophrenia helps to communicate that antipsychotic medications are not the *de facto* treatment of catatonia. Antipsychotic medications can often worsen catatonia, precipitate neuroleptic malignant syndrome, or introduce extrapyramidal signs that confound a reliable assessment of catatonia. Whereas the use of antipsychotic medications may be an appropriate part of managing catatonia associated with a psychotic disorder, general caution is advised in catatonia [28]. *ICD-11* allows specific treatment for catatonia with benzodiazepines and electroconvulsive therapy (ECT) to be started without having to treat a patient as if they have schizophrenia.

## Outstanding issues

Which features (*n.b.*, we use the term ‘features’ to apply to signs and symptoms, though most features in catatonia are properly signs) should be included among potential diagnostic criteria and which ones should be grouped remains a pressing question. As shown in Table 2, *DSM-5-TR* lists 12 eligible features whereas *ICD-11* lists 15 and, although they are broadly aligned, there are distinctions. Unlike *DSM-5-TR*, *ICD-11* includes staring, ambitendency, verbigeration, and rigidity, which are classic catatonic signs not recognized among *DSM-5-TR* criteria. It also takes a more expansive view of hyperkinetic catatonia by also including impulsivity and combativeness in terms of what qualifies for increased psychomotor activity; however, from a practical perspective, clinically significant impulsivity or combativeness would already qualify for hyperactivity in *DSM-5-TR*.

As for the question of combining vs. splitting, *ICD-11* groups echolalia and echopraxia as echophenomena whereas *DSM-5-TR* separates these. This distinction between sets of diagnostic criteria raises an interesting consideration regarding the inconsistent handling of analogous behavioural and speech findings in catatonia. For instance, immobility and mutism represent diminution of behaviour and speech, respectively, but are distinct criteria; similarly, stereotypy and verbigeration (i.e., verbal stereotypy) are listed separately. However, echophenomena are currently combined in *ICD-11*, partly due to pragmatic considerations, in that echophenomena are quite rare. Conversely, mannerisms are described in *ICD-11* as ‘odd, purposeful movements’, so they do not encompass speech, although speech that is ‘robotic’, ‘theatrical’, or ‘peculiar’ is sometimes considered manneristic [73–75] and the index to *DSM-5* describes a mannerism as ‘a peculiar and characteristic individual style of movement, action, thought, or speech’. This outcome in *ICD-11* is the result of the way in which these clinical features have been dissected in the existing literature. Future research needs to establish whether there is value in combining or separating clinical features such as stereotypies/verbigeration, immobility/mutism, echopraxia/echolalia and mannerisms across verbal and motor domains.

The threshold for diagnosis also remains an active question. For instance, Max Fink, among others, has long supported a lower threshold for catatonia in terms of the number of clinical features but has required that a diagnosis be validated by treatment with benzodiazepines or ECT [76]. Using lorazepam, another benzodiazepine, or zolpidem is sometimes recommended to assist with diagnosis of catatonia as a form of biological validation [28, 77, 78]. It is possible that such a challenge may be included in future diagnostic manuals, though it should be noted that clinical response to benzodiazepines or ECT is not specific to catatonia; additionally, there are less common causes of catatonia (typically in the setting of a primary medical or neurologic disorder) that do not respond to traditional treatment approaches. There is also the risk of (1) circular reasoning if a condition is defined in terms of its recommended treatment and (2) failing to recognize catatonia refractory to benzodiazepines or ECT.

A key reason why the threshold for diagnosis of catatonia remains at 3 features is because this has been shown to represent the best balance between sensitivity and specificity [79, 80]. However, we recognize that an *ICD-11* diagnosis of catatonia will not achieve a specificity of 100%, as it will likely capture some individuals who do not have catatonia based on a theoretical gold standard. This may be more relevant for certain clinical populations, such as those with delirium, where a higher number of catatonic features has been suggested [15], or neurodevelopmental disorders, where some specialists have advised subtracting baseline features from the current presentation in scoring catatonia [64]. Notwithstanding adjustments in certain populations, it is still likely that current definitions of catatonia will capture some of the boundary conditions mentioned in Table 4.

The threshold question also applies to the individual features of catatonia. These could be defined more specifically in terms of the nature of presentation, duration and severity [81]. Clinical instruments vary, occasionally quite widely, in what alteration qualifies for each feature. The *ICD-11* provides descriptions very similar to that of *DSM-5-TR*; however, no feature is formally operationalized. Indeed, this was among the primary reasons for developing the Bush-Francis Catatonia Rating Scale (BFCRS) Training Manual and Coding Guide, which also distinguishes between an interval assessment (e.g. over a 24 h period) and a state assessment (i.e. cross-sectional) [82]. Other catatonia rating scales exist, including the Rogers Catatonia Scale [59], the Northoff Catatonia Rating Scale [83], the Brӓunig Catatonia Rating Scale [84] and the Kanner Scale [85], but there are differences between scales in their definition of individual catatonic features [81]. It is likely that various thresholds are appropriate in different contexts: research studies may take a more restrictive definition of catatonia than clinical practice and catatonia in the context of certain comorbidities, such as delirium or neurodevelopmental conditions, may require a modified definition.

Biomarkers for catatonia have been identified and include functional neuroimaging [86], serum iron and creatine kinase [87] and electroencephalography [88]. If the sensitivity of such biomarkers is optimised, they could be useful for screening, while a high specificity could make them useful for diagnosis of catatonia.

Finally, any diagnostic classification is only as good as its clinical use. While *ICD-11* does much to advance and develop the concept of catatonia, it remains to be seen how much these changes will be translated into clinical practice and the extent to which clinical practice improves as a result.

## Conclusion

*ICD-11* represents a substantial improvement on previous clinical classifications of catatonia in *ICD*. It takes into consideration the burgeoning evidence on catatonia outside of schizophrenia, harmonises criteria across underlying conditions, and removes the *de facto* requirement to suffer from catatonia for at least a month to meet diagnostic criteria. Future classifications should be informed by research on the overall thresholds for the condition as well as the detailed definitions of individual clinical features.

## Data Availability

No datasets were generated or analysed during the current study.

## Abbreviations

BFCRS: Bush–Francis Catatonia Rating Scale
DSM: Diagnostic and Statistical Manual of Mental Disorders
ECT: Electroconvulsive therapy
ICD: International Statistical Classification of Diseases and Related Health Problems
NIHR: National Institute for Health and Care Research
